# Complete Genome Sequence of *Lactobacillus plantarum* CGMCC 8198

**DOI:** 10.1128/genomeA.01559-16

**Published:** 2017-02-09

**Authors:** Qing-Qing Dong, Hai-Jie Hu, Xue-Gang Luo, Qiu-Tong Wang, Xiang-Chao Gu, Hao Zhou, Wen-Juan Zhou, Xiao-Meng Ni, Tong-Cun Zhang

**Affiliations:** a Key Lab of Industrial Fermentation Microbiology, Tianjin University of Science & Technology; Ministry of Education, Tianjin, People’s Republic of China; b Tianjin Key Lab of Industrial Microbiology, College of Biotechnology, Tianjin University of Science & Technology, Tianjin, People’s Republic of China; c Tianjin Institute of Industrial Biotechnology, Chinese Academy of Sciences, Tianjin, People’s Republic of China

## Abstract

We report the complete genome sequence of *Lactobacillus plantarum* CGMCC 8198, a novel probiotic strain isolated from fermented herbage. We have determined the complete genome sequence of strain *L. plantarum* CGMCC 8198, which consists of genes that are likely to be involved in dairy fermentation and that have probiotic qualities.

## GENOME ANNOUNCEMENT

Lactic acid bacterium-fermented products, such as yogurt and cheese, are popular foods in the world. *Lactobacillus plantarum* is a type of versatile lactobacillus that has been extensively characterized functionally to document its probiotic attributes (1–6). The strain *L. plantarum* CGMCC 8198 (formerly named *L. plantarum* TH1) was isolated in our previous study (7, 8). It has been shown that *L. plantarum* CGMCC 8198 has high bile salts resistance and hypocholesterolemic effects in mice. In the present study, the complete genome sequence of *L. plantarum* CGMCC 8198 was determined by whole-genome shotgun sequencing using Illumina technology. The genome was assembled using Spades (9) and Edena (10) software, and multiplex PCR was used to close the gaps and remove regions of low coverage (11). The software program Glimmer (12) and the RAST suite (13) were used to identify protein-coding genes and for gene annotation, respectively.

The complete genome of strain *L. plantarum* CGMCC 8198 contains a single circular chromosome of 3,086,220 bp. The overall G+C content of the chromosome is 36.8%, with 2,908 predicted open reading frames (ORFs), of which 2,388 were functionally classified. Besides, 2,908 tRNA-coding genes and 3,086,220 rRNA-coding genes were also found (14).

A comparative analysis of strains *L. plantarum* CGMCC 8198 and *L. plantarum* JDM1 was performed using blast2go. The sequencing results were compared with the reference sequence and a series of SNP loci was obtained. A comparative genomics approach was also performed to analyze the biosynthesis metabolic pathways of amino acid, glycan, and carbohydrate, and the genes involved in the biosynthesis of other secondary metabolites, cell motility, infectious bacterial diseases, and bile salts stress proteins. The results show that this strain can not only hydrolyze extracellular proteins, transport, and perform enzymolysis efficiently, but has more complete enzyme systems of transamination and the deamination pathway. In addition, bile salt hydrolase (BSH) activity has been reported in *Bifidobacterium* (15, 16), *Lactobacillus* (17), *Enterococcus* (18), *Bacteroides* (19), and many other bacteria. However, data on the BSH enzymes from* L. plantarum* are limited and the precise mechanism underlying the hypocholesterolemic effect of BSHs is still not clear. Our analysis found that five putative active sites, Cys1, Asp20, Tyr78, Asn171, and Arg224, were found in all three bsh genes of *L. plantarum* CGMCC 8198. However, these active sites were the same as in PVA but were a little different with BSHs in *Lactobacillus*. For example, the catalytic residue of Tyr78 was replaced by Asn78 in BSHs of *L. johnsonii* ATCC 33200 and *L. acidophilus*. These results indicate that BSHs in* L. plantarum* CGMCC 8198 might have individual mechanisms for the hydrolysis of bile salts, and might escape cell death by bile salts in the intestine and reduce plasma cholesterol *in vivo* through the hydrolysis activity of its BSHs. This work indicates that this novel probiotic stain could be used in the food or drug system to improve the health of patients suffering from cholesterol-related diseases.

### Accession number(s).

The complete genome of *L. plantarum* CGMCC 8198 has been deposited in GenBank under accession no. MEGY00000000.
